# Real-life experience with the specific reversal agent idarucizumab for the management of emergency situations in dabigatran-treated patients: a series of 11 cases

**DOI:** 10.1007/s11239-017-1476-2

**Published:** 2017-02-16

**Authors:** Milan R. Vosko, Christof Bocksrucker, Rafał Drwiła, Petr Dulíček, Tomas Hauer, Johannes Mutzenbach, Christoph J. Schlimp, David Špinler, Thomas Wolf, Daša Zugwitz

**Affiliations:** 1 Department of Neurology 2, Kepler Universitätsklinikum, Med Campus III, Krankenhausstr. 9, 4020 Linz, Austria; 2 Department of Neurology, KonventhospitalBarmherzige Brueder Linz, Linz, Austria; 3 Department of Anesthesiology and Intensive Care, John Paul II Hospital, Medical College of Jagiellonian University, Krakow, Poland; 4 Fourth Department of Internal Medicine, Hematology, Hradec Králové Faculty of Medicine, Hradec Králové University Hospital, Hradec Králové, Czech Republic; 5 Department of Internal Medicine, České Budějovice Regional Hospital, and Faculty of Health and Science, University of South Bohemia, České Budějovice, Czech Republic; 6 Department of Neurology, Christian Doppler Medical Center, Paracelsus Medical University, Salzburg, Austria; 7 Department of Anesthesiology and Intensive Care, AUVA Trauma Hospital, Klagenfurt, Austria; 8 Department of Internal Medicine, Ústí nad Orlicí Hospital, Ústí nad Labem, Czech Republic; 9 Department of Cardiology, Pardubice Regional Hospital, Pardubice, Czech Republic; 10 Department of Neurology, Wiener Neustadt Regional Hospital, Wiener Neustadt, Austria; 11 General Hospital Jesenice, Jesenice, Slovenia

**Keywords:** Direct oral anticoagulants, Non-vitamin K antagonist oral anticoagulants, Non-valvular atrial fibrillation, Critical bleeding, Emergency procedures, Anticoagulation reversal

## Abstract

Non-vitamin K antagonist oral anticoagulants (NOACs) have a favorable benefit-risk profile compared with vitamin K antagonists. However, the lack of specific reversal agents has made the management of some patients receiving long-term treatment with NOACs problematic in emergency situations such as major bleeding events or urgent procedures. Idarucizumab, a fully humanized Fab antibody fragment that binds specifically and with high affinity to dabigatran, was recently approved for use in adult patients treated with dabigatran when rapid reversal of its anticoagulant effect is required. Clinical experience with idarucizumab is currently limited. We report 11 real-life clinical cases in which idarucizumab was used after multidisciplinary consultation in a variety of emergency situations including severe postoperative bleeding, emergency high-bleeding-risk surgery (hip/spine surgery and neurosurgery), invasive diagnostic testing (lumbar puncture), intracranial bleeding (pre-pontine subarachnoid hemorrhage and lobar intracerebral hemorrhage) and thrombolysis with recombinant tissue plasminogen activator for acute ischemic stroke. This case series illustrates the role of idarucizumab in improving patient safety in rare emergency situations requiring rapid reversal of the anticoagulant effect of dabigatran, while highlighting the importance of information and education about the availability and appropriate use of this recently approved specific reversal agent.

## Introduction

Non-vitamin K antagonist oral anticoagulants (NOACs) have been developed as direct and specific inhibitors of thrombin (dabigatran) or factor Xa (rivaroxaban, apixaban and edoxaban) in order to overcome the limitations of vitamin K antagonists [1]. Although NOACs have a favorable benefit-risk profile compared with vitamin K antagonists [2], and are associated with similar or better outcomes in case of major bleeding [3] and urgent procedures [4], the lack of specific reversal agents has made the management of some patients receiving long-term treatment with NOACs problematic in rare emergency situations and has been perceived as a barrier to the more widespread adoption of NOACs in clinical practice [5].

Idarucizumab, a humanized Fab antibody fragment that binds specifically and with high affinity to dabigatran [6], has been approved in the United States and Europe for use in adult patients treated with dabigatran when rapid reversal of its anticoagulant effect is required. The efficacy and safety of idarucizumab (5 g administered intravenously as two 2.5-g/50-mL infusions or as a bolus injection) in dabigatran-treated patients with uncontrollable or life-threatening bleeding and in those requiring an urgent surgical or invasive procedure are being investigated in RE-VERSE AD, a global phase 3 prospective cohort study [7]. An interim analysis including the first 90 patients enrolled in the study (51 with serious bleeding and 39 requiring an urgent procedure) reported rapid, complete and sustained reversal of the anticoagulant effect of dabigatran following idarucizumab administration [8].

Clinical experience with the use of idarucizumab for urgent reversal of the anticoagulant effects of dabigatran is currently limited. For the first time, we report 11 cases in which idarucizumab was used in emergency situations after multidisciplinary consultation in real-life clinical practice. These cases were presented and discussed during an expert meeting held in Vienna in July 2016. In the meantime, two of these cases (No. 1 and No. 11) have been reported in separate publications [9, 10]. They are included in this series to reflect the variety of clinical situations discussed during the expert meeting.

### Case series presentation

Selected information on patients’ conditions, indications for the use of idarucizumab, coagulation test results and clinical outcomes is provided in Table 1.

**Table 1:** Selected information on patients’ conditions, indications for the use of idarucizumab, coagulation test results and clinical outcomes

Patient presentation	Idarucizumab administration	Coagulation tests		Clinical outcome
		Before idarucizumab administration	After idarucizumab administration	
83-year-old male *Cause of hospital admission*/*diagnosis* Ascending aortic aneurysm complicated by acute aortic syndrome (type A intramural hematoma) *Dabigatran treatment* NVAF 110 mg b.i.d. Last intake on the day of admission Stage 3 chronic kidney disease Serum creatinine: 264 µmol/L eGFR: 19 mL/min/1.73 m^2^ Hb: 104 g/L PLT count: 129 x 10^9^/L	*Reason for administration* Excessive bleeding after emergency cardiac surgery (supracoronary ascending aortic and hemiarch replacement) *Time of administration* Following CPB cessation *Mode of administration* 5-minute intravenous infusion (5 g)	dTT: 209 ng/mL	dTT: <32 ng/mL	Discharged to a local hospital for further management on postoperative day 8; uneventful follow-up on postoperative day 30
93-year-old female *Cause of hospital admission* Periprosthetic femoral hip fracture due to a fall *Dabigatran treatment* NVAF 110 mg b.i.d. Last intake on the day of admission Serum creatinine: 0.88 mg/dL Hb: 133 g/L PLT count: 192 x 10^9^/L	*Reason for administration* Urgent orthopedic surgery *Time of administration* Before surgery (same day) *Mode of administration* Intravenous infusion (2 × 2.5 g)	TT: 170 s aPTT: 40 s	TT: 17 s aPTT: 28 s	Postoperative monitoring in the ICU for 4 days with no complications; transfer to geriatric rehabilitation
70-year-old male *Cause of hospital admission*/*diagnosis* Comminuted spine fracture *Dabigatran treatment* NVAF 150 mg b.i.d. Last intake on the day of admission CrCl: 60 mL/min	*Reason for administration* Worsening condition requiring spine surgery *Time of administration* Before surgery (same day), 3 days after last dabigatran intake *Mode of administration* 10-minute infusion (5 g)	dTT: 54 ng/mL TT: 95 s aPTT: 44.3 s	dTT: 0 ng/mL TT: 18 s aPTT: 31.4 s	Successful surgery with no complications
78-year-old male *Cause of hospital admission* Collapse in public and acute onset of left-sided weakness *Diagnosis* Left middle cerebral artery occlusion and compensated critical cerebral ischemia *Dabigatran treatment* Persistent NVAF 150 mg b.i.d. Last intake on the day of admission Hb: 44.7 g/L	*Reason for administration* Emergent extracranial-intracranial bypass surgery *Time of administration* Immediately before surgery *Mode of administration* Intravenous infusion (5 g)	TT: >120 s aPTT: 47 s	TT: 18.4 s aPTT: 84.9 s	Successful surgery without complications; no neurological deficit except for very mild weakness of acral parts of lower extremities
87-year-old male *Cause of hospital admission* General malaise and weakness *Diagnosis* Subdural hematoma and brain hernia *Dabigatran treatment* Chronic NVAF 110 mg b.i.d. Last intake 7 hours before admission Serum creatinine: 119 µmol/L eGFR: 52 mL/min/1.73 m^2^ Hb: 151 g/L PLT count: 182 x 10^9^/L	*Reason for administration* Neurosurgery planned the next day *Time of administration* 9 h after last dabigatran intake *Mode of administration* Fast intravenous infusion (5 g)	dTT: 168 ng/mL TT: unmeasurable aPTT: 49.9 s	dTT: 20 ng/mL TT: 19.2 s aPTT: 28.4 s	Discharged after 5 days with good neurological outcome
81-year-old female *Cause of hospital admission* Somnolence and meningeal symptoms *Dabigatran treatment* NVAF 110 mg b.i.d. Last intake on the day of admission CRP: 4.4 mg/dL	*Reason for administration* Lumbar puncture indicated due to suspicion of neuroinfection *Time of administration* Immediately before the procedure *Mode of administration* 10-minute intravenous infusion (5 g)	aPTT: 32.8 s	No coagulation tests performed	Lumbar puncture performed with no bleeding complication; neuroinfection disproved and drug (opiate) intoxication diagnosed
44-year-old woman *Cause of hospital admission* Sudden headache (in the evening); nausea and sickness *Neurological examination* Meningism, no focal neurological deficit *Imaging* CT scan reveals pre-pontine subarachnoid hemorrhage *Dabigatran treatment* DVT 150 mg b.i.d. Last intake on the day of admission (8:00 in the morning) CRP: 0.3 mg/dL WBC count: 10.3 × 10^9^/L	*Reason for administration* Anticoagulation reversal indicated following diagnosis of subarachnoid hemorrhage *Time of administration* 2 h after diagnosis, 4 h after onset of symptoms *Mode of administration* Fast infusion (5 g)	TT: 73.9 s aPTT: 39 s	TT: 16.7 s aPTT: 33 s	Patient discharged after follow-up imaging (no aneurysm, no vasospasm and no intracerebral hemorrhage)
88-year-old man *Cause of hospital admission* Acute onset of high-graded left-sided hemiparesis *Imaging* MRI and CT reveal parenchymal hemorrhage in the right temporo-parietal lobe *Dabigatran treatment* NVAF 110 mg b.i.d. Last intake 2–4 h prior to admission CrCl (Cockcroft-Gault): 61 mL/min	*Reason for administration* Anticoagulation reversal indicated following diagnosis of intracerebral hemorrhage *Time of administration* 35 min after admission *Mode of administration* Intravenous infusion (5 g)	TT > 150 s dTT: 154 ng/mL	TT < 32 s dTT < 20 s	Patient transferred to a normal neurological ward in stable condition
78-year-old man *Cause of hospital admission* Speech disorder noticed by spouse in the morning; aphasia, difficulty walking, right faciobrachial hemiparesis *Diagnosis* MRI revealed beginning of ischemia and presence of microbleeds *Dabigatran treatment* NVAF 110 mg b.i.d. Last intake on the day admission Serum creatinine: 1.1 mg/dL eGFR: 68.8 mL/min/1.73 m^2^ Hb: 147 g/L PLT count: 175 x 10^9^/L	*Reason for administration* Decision to perform thrombolysis with rt-PA *Time of administration* 20 min before rt-PA infusion *Mode of administration* Intravenous bolus injection (5 g)	dTT: 134 ng/mL TT: >150 s aPTT: not available	dTT: <32 ng/mL TT: 22.9 s aPTT: 24.6 s	Thrombolysis with rt-PA (reduced dose 0.6 mg/kg) was performed without complications
84-year-old man *Cause of hospital admission* Right-sided hemiparesis *Neurological examination* Aphasia, right-sided hemiparesis (NIHSS 9, mRS 5) Imaging: brain CT: ASPECTS 10, brain MRI: restricted diffusion on diffusion-weighted imaging in basal ganglia and cella media left *Dabigatran treatment* NVAF 110 b.i.d. Last intake on the day of admission Serum creatinine: 1.46 mg/dL eGFR: 49 mL/min/1.73 m^2^ Hb: 125 g/L PLT count: 154 x 10^9^/L	*Reason for administration* Decision to perform thrombolysis *Time of administration* 20 min before rt-PA infusion, 3.5 h after last dabigatran intake *Mode of administration* Intravenous infusion (5 g)	dTT: 79 ng/mL TT: 129 s aPTT: 41.6 s	dTT: < 20 ng/mL TT: 16 s aPTT: 31.5 s	Thrombolysis with rt-PA (70 mg) was performed without complications Patient improved to an NIHSS of 4 and was sent to a rehabilitation unit on day 9
68-year-old man *Cause of hospital admission* Visual disturbances, dizziness and slight headache *Neurological examination* Right-sided homonymous hemianopsia (mRS 3) *Imaging* Brain MRI revealed restricted diffusion on diffusion-weighted imaging in the medial part of the left occipital lobe without demarcation in FLAIR *Dabigatran treatment* NVAF 110 mg b.i.d. Last intake on the day of admission Serum creatinine: 0.76 mg/dL CrCl: 102 mL/min Fibrinogen: 547 mg/dL	*Reason for administration* Decision to perform thrombolysis with rt-PA *Time of administration* 25 min before rt-PA infusion and ~2 h after last dabigatran intake *Mode of administration* Fast intravenous infusion (5 g)	aPTT: 34 s	aPTT: 33 s	Transcranial Doppler sonography shortly after rt-PA infusion: regular blood flow in both posterior cerebral arteries. Patient discharged on day 3 with slightly improved hemianopsia (NIHSS 3, mRS 2)

*aPTT* activated partial thromboplastin time, *b.i.d*. twice daily, *CrCl* creatinine clearance, *CRP* C-reactive protein, *dTT* dilute thrombin time, *DVT* deep vein thrombosis, *eGFR* estimated glomerular filtration rate, *Hb* hemoglobin concentration, *ICU* intensive care unit, *mRS* modified rankin scale, *NIHSS* National Institutes of Health Stroke Scale, *NVAF* non-valvular atrial fibrillation, *PLT* platelet, *rt-PA* recombinant tissue plasminogen activator, *TT* thrombin time

#### Case 1: an 83-year-old man with excessive bleeding after emergency cardiac surgery [9]

An 83-year-old man presented with ascending aortic aneurysm complicated by acute aortic syndrome [type A intramural hematoma (IMH)], confirmed by computer tomography. The condition, in the absence of a surgical intervention, is associated with a poor prognosis (morality rate of 1% per hour during the first 48 h). Transthoracic echocardiography identified moderate aortic insufficiency and acute exacerbation of chronic kidney disease [estimated glomerular filtration rate (eGFR) of 19 mL/min/1.73m²]. The patient had non-valvular atrial fibrillation (NVAF) for which he was on dabigatran (110 mg b.i.d., last intake on the day of admission). Furthermore, he had stage 3 chronic kidney disease, arterial hypertension, and a history of right hemispheric ischemic stroke, peripheral arterial disease, peptic ulcer disease of duodenum and basal cell carcinoma resection (face and chest).

Emergency cardiac surgery with cardiopulmonary bypass (CPB) and deep hypothermia with temporary circulatory arrest was performed. The dabigatran level [dilute thrombin time (dTT)] before surgery was 209 ng/mL. Anticoagulation for the intervention was achieved by administration of heparin (500 IU/kg) before the onset of CPB and monitored using the activated clotting time with a target of 400 s during CPB. The supracoronary ascending aortic and hemiarch replacement procedure was successfully performed. The aortic cross-clamping time was 64 min, cerebral perfusion time was 34 min and total CPB time was 195 min. At the end of CPB, anticoagulation was reversed by protamine to obtain a normal activated clotting time.

Tranexamic acid was administered in two doses [20 mg/kg intravenously (i.v.) after sternotomy and 20 mg/kg i.v. after the end of CPB). At the end of CPB, the hemoglobin level was 8.4 g/dL. A total of 12 units of platelet concentrate and 3 units of fresh frozen plasma were administered in the operating room. Due to excessive perioperative bleeding, idarucizumab (5 g i.v.) was administered following CPB cessation. After surgery, the patient was transferred to the intensive care unit (ICU) for postoperative ventilation and extubated after 17 h. The level of dabigatran after idarucizumab administration was below 32 ng/mL. Three units of packed red blood cells (PRBC) were transfused in the intensive care unit (ICU). Total postoperative drainage was 470 mL.

During the postoperative course, the patient required diuretic treatment and intensive pulmonary rehabilitation. The postoperative course was complicated with pneumonia which resolved after antibiotics. Anticoagulation with warfarin was reinitiated, and on postoperative day 8 the patient was discharged to a local hospital for further management. On postoperative day 30, the follow-up was uneventful.

#### Case 2: a 93-year-old woman requiring urgent treatment of a periprosthetic femoral hip fracture

After a fall, a 93-year-old woman was admitted to hospital with a periprosthetic femoral hip fracture and bleeding caused by the accident. The patient had previously been prescribed dabigatran 110 mg b.i.d. for NVAF and had an implanted pacemaker. The laboratory results at admission included an activated partial thromboplastin time (aPTT) of 46 s and a thrombin time (TT) of 225 s. In the next 24 h, TT (170 s) did not fall to acceptable values for surgery and the patient was transfused 2 units of PRBC due to blood loss into her thigh. Taking into consideration a national recommendation to perform surgery of fractures next to the hip within 48 h, the interdisciplinary decision was made to perform revision arthroplasty the next day and to administer the first vial of idarucizumab (2.5 g i.v.) before the procedure, which resulted in a TT of 17 s.

Surgery was initiated, and during the operation the patient received the second vial of idarucizumab (2.5 g i.v.) as well as 1 g of tranexamic acid, 2 g of fibrinogen concentrate, 15 µg of desmopressin, 5 units of PRBC (including autologous cell salvaged blood) and 2 units of solvent/detergent plasma. The patient remained under postoperative monitoring in the intensive/intermediate care unit for 4 days without any complication. On postoperative days 1 and 2, TT rose again to 129 s and 131 s, respectively, after which it fell back to 49 s on day 3. Postoperative thromboprophylaxis was initiated with dalteparin (2 × 5000 IU) on day 3. The patient was then transferred to a normal ward, where dabigatran was reinitiated by the internist, and then to geriatric rehabilitation.

#### Case 3: a 70-year-old man with a comminuted spine fracture requiring surgery

A 70-year-old man was admitted with a comminuted spine fracture (L1 type A4 and posterior elements of L2). The medical history of the patient showed a number of comorbidities including NVAF, coronary artery disease, apex aneurysm and hyperlipoproteinemia. Among multiple medications, the patient was prescribed dabigatran 150 mg b.i.d. (he had taken the last dose on the morning of admission day).

The patient’s condition was assessed as relatively stable. After the first laboratory results showed an aPTT of 63.2 s, surgery was planned 3 days later to allow time for the elimination of dabigatran. On the day surgery was planned, i.e. 3 days after dabigatran treatment was discontinued, laboratory tests showed a dTT of 54 ng/mg, a TT of 95 s and an aPTT of 44.3 s, and the patient’s condition was worsening. The decision was made to administer idarucizumab (5 g i.v.) and the surgical procedure took place without complications. Laboratory results after surgery showed a dTT of 0 ng/mL, a TT of 15 s and an aPTT of 31.4 s.

#### Case 4: a 78-year-old man with left middle cerebral artery occlusion and compensated critical cerebral ischemia undergoing emergent extracranial-intracranial bypass surgery

A 78-year-old man was referred to a comprehensive stroke center 60 min after displaying an acute onset of left-sided weakness and collapsing in public. No obvious neurological deficit could be detected. The patient had persistent NVAF (treated with dabigatran 150 mg b.i.d.), arterial hypertension and a history of deep vein thrombosis.

Multimodal CT scan showed a left middle cerebral artery occlusion and compensated critical cerebral ischemia, which caused the stroke. Thrombolytic therapy was excluded due to the anticoagulant effect of dabigatran (laboratory results showed a TT > 120 s and an aPTT of 47 s). Mechanical thrombectomy could not be performed due the kinking of the right common carotid artery and stenosis of the right internal carotid artery. A conservative approach was rejected due to an extremely high risk of neurological deterioration.

The decision was made to perform emergent extracranial-intracranial bypass surgery after administration of idarucizumab. As the reversal agent was unavailable on site, urgent transport was arranged from another hospital. Ten minutes after the patient was administered 5 g of idarucizumab, TT was 17.8 s and the patient was transferred to the operating room. One hour later, a new blood sample showed a TT of 18.4 s and coagulation assessed by ROTEM was normal, allowing continuation of the procedure.

The surgery was successful without complications and the patient was extubated shortly after the procedure. There was no complication and no neurological deficit except for very mild weakness of acral parts of lower extremities. Dabigatran treatment was resumed on postoperative day 7. The patient has fully recovered and is able to take care of his disabled wife.

#### Case 5: an 87-year-old man with a subdural hematoma and a brain hernia requiring neurosurgery

A usually active and healthy 87-year-old man presented to the emergency department with general malaise and weakness that had started 5 days before. His condition worsened on the day of admission with sleepiness, occasional incoherent speech, confusion and lack of lateralization, as well as a tremor of both hands. The chest sounds were normal; an arrhythmia was found, but no edema. The patient was being treated for arterial hypertension and NVAF for which he was prescribed dabigatran 110 mg b.i.d. He had no known allergies.

A CT scan revealed a large subdural hematoma and brain herniation across the falx and tentorium cerebelli. Although the patient was quite active and often rode a bicycle, no obvious evidence of a fall and no external sign of trauma was found. After consultation with the neurosurgeon, surgery was planned the next day.

The laboratory results showed moderate renal impairment reflected by an eGRF of 52 mL/min/1.73 m². The presence of dabigatran was confirmed by a dTT of 168 ng/mL. Approximately 9 h after the last intake of dabigatran and 7 h after admission, the patient received a fast i.v. infusion of 5 g idarucizumab, during and after which the condition of the patient was stable.

The following morning, 15 h after idarucizumab administration, dTT had decreased to levels under 20 ng/mL. The patient was transferred to neurosurgery and trepanation was performed. Five days later, the patient was discharged home with normal neurological status. Anticoagulation therapy was withheld with a recommendation for further evaluation before treatment resumption.

#### Case 6: an 81-year-old woman requiring lumbar puncture due to a suspicion of neuroinfection

An 81-year-old woman was referred to hospital because of somnolence and meningeal symptoms. She was treated for NVAF (for which she took dabigatran 110 mg b.i.d.) and diabetes mellitus, and she had a history of intracranial hemorrhage. Laboratory tests revealed a CRP of 4.4 mg/dL, leading to suspicion of a neuroinfection.

A lumbar puncture was urgently needed to confirm or rule out the suspected neuroinfection, but the procedure is contraindicated in the presence of anticoagulation therapy. As the last intake of dabigatran had taken place in the morning and coagulation tests were prolonged, idarucizumab (5 g i.v.) was administered to reverse the anticoagulation activity of dabigatran. The lumbar puncture was performed 30 min later without additional laboratory tests. There was no bleeding complication and neuroinfection was disproved: the patient actually suffered from an opiate intoxication. Dabigatran treatment was reinitiated the next day.

#### Case 7: an 44-year-old woman with a pre-pontine subarachnoid hemorrhage

A 44-year-old woman was admitted to hospital due to a sudden and violent headache (estimated 10 on Visual Analog Scale) with nausea and sickness. She presented with meningism but no focal neurological deficit. She was treated for hypothyroidism and Conn syndrome, and her medical history included a stroke of the right hemisphere and a benign liver tumor. She was on dabigatran 150 mg b.i.d. for the treatment of deep vein thrombosis, with the last intake at 8:00 in the morning on the day of admission.

A CT scan revealed a pre-pontine subarachnoid hemorrhage. No specific cause was identified, but the patient was fragile and had multiple comorbidities. Laboratory tests showed a TT of 73.9 s and an aPTT of 39 s. Idarucizumab was not available locally but was obtained from another hospital. The patient was administered idarucizumab (5 g i.v.) in the late evening, and the following morning the TT had decreased to 16.7 s. The patient was discharged 9 days later after follow-up imaging showed no aneurysm, no vasospasm and no evidence of intracerebral hemorrhage.

#### Case 8: a 78-year-old man with aphasia, difficulty walking and right faciobrachial hemiparesis undergoing thrombolysis for acute ischemic stroke

A 78-year-old man was admitted to hospital in the morning after his wife noticed aphasia, difficulty walking and right faciobrachial hemiparesis. MRI revealed recent-onset ischemia and the presence of microbleeds. The patient was treated with dabigatran 110 mg b.i.d. for NVAF, with the last intake the morning of admission day.

Thrombolysis with recombinant tissue plasminogen activator (rt-PA) was indicated. However, laboratory tests showed a dTT of 134 ng/mL. It was thus decided to administer an intravenous bolus injection of idarucizumab (5 g). Thrombolysis was performed 20 min later without complications (a reduced dose of rt-PA, 0.6 mg/kg, was used). Approximately 30 min after idarucizumab administration, the dTT was below 32 ng/mL and remained under this detection level during the next 3 days. The patient recovered fully from his stroke and was able to go back to a very active way of life.

#### Case 9: an 88-year-old man with spontaneous intracerebral hematoma in the right temporo-parietal lobe

An 88-year-old man with the history of a NVAF taking dabigatran 110 mg b.i.d was admitted to the hospital due to acute onset of high-graded left-sided hemiparesis. The initial NIHSS score was of 10. Cerebral imaging with MRI and CT revealed parenchymal hemorrhage in the right temporo-parietal lobe. The following laboratory test results were obtained: creatinine clearance (Cockcroft-Gault) 61 mL/min, TT > 150 s and dTT 154 ng/mL. The last intake of dabigatran was reported to have taken place 2–4 h prior to admission. Acute reversal of the anticoagulation effect of dabigatran was performed with 5 g of idarucizumab within 35 min after admission. Shortly after administration of idarucizumab, dTT decreased below 32 ng/mL and TT below 20 s; the values remained low for 4 consecutive days. The size of the intracranial hematoma remained unchanged. The patient was transferred to a normal neurological ward on day 7 in stable condition.

#### Case 10: an 84-year-old man with right-sided hemiparesis and aphasia undergoing thrombolysis for acute ischemic stroke

An 84-year-old male patient was admitted to the stroke unit at 11:00 in the morning because of right-sided hemiparesis and aphasia. Symptoms had started 3 h prior to admission. The patient was on dabigatran 110 mg b.i.d. because of NVAF. The lower dose regimen had been selected considering the patient’s age (above 80 years) and his slightly elevated serum creatinine level (1.46 mg/dL). The last dabigatran dose had been taken on the morning of admission.

The initial NIHSS score was 9 and cerebral CT was normal, providing an ASPECTS score of 10. An ischemic stroke involving the basal ganglia and the cella media was diagnosed. Laboratory tests showed a dabigatran level of 79 ng/mL and a TT of 129 s. As no other contraindications to thrombolysis with rt-PA were identified, idarucizumab (5 g) as an intravenous infusion was administered to reverse the anticoagulant effects of dabigatran.

After administration of idarucizumab, a blood sample was taken and thrombolysis with the standard dose of rt-PA was immediately initiated. Laboratory tests showed a dabigatran level below 20 ng/mL and a TT of 16 s. Thrombolysis was successfully completed. Treatment with dabigatran 110 mg b.i.d. was restarted on day 2. The patient’s condition improved and on day 9 he was admitted to a rehabilitation unit with an NIHSS score of 4.

#### Case 11: a 68-year-old man with undergoing thrombolysis for a right-sided homonymous hemianopsia [10]

A 68-year-old man present at hospital after a sudden onset of visual disturbances, dizziness and slight headache. Neurological examination 25 min later revealed right-sided homonymous hemianopsia and marginal evidence of non-fluent aphasia from a stroke 20 months earlier. Brain MRI revealed restricted diffusion on diffusion-weighted imaging in the medial part of the left occipital lobe without demarcation in FLAIR.

The patient’s medical history included recurrent transient ischemic attacks. He had been taking dabigatran 110 mg b.i.d. for NVAF. At the time of treatment initiation, his renal function was slightly impaired; however, the prescribed dose regimen may have been inappropriate given the patient’s age and medical history. Laboratory examinations revealed an eGFR of 102 mL/min, a dTT of 34 ng/mL and an aPTT of 34 s. It was decided to neutralize the anticoagulant activity of dabigatran and to perform thrombolysis with rt-PA, considering hemianopsia as a functionally relevant deficit (mRS of 3).

The patient received a rapid i.v infusion of idarucizumab (5 g), which was followed, after 10 min (and a total of 110 min from symptom onset), by intravenous thrombolysis with rt-PA (70 mg). Transcranial Doppler sonography shortly after rt-PA infusion showed regular blood flow in both posterior cerebral arteries. Transthoracic echocardiography on day 2 did not reveal cardiac thrombi or structural heart disease. The patient was discharged on day 3 with slightly improved hemianopsia (NIHSS 3, mRS 2). Dabigatran treatment was resumed at the higher dosage (150 mg b.i.d.) as renal function tests were normal.

## Discussion

We report 11 real-life clinical cases in which idarucizumab was used after multidisciplinary consultation in a variety of emergency situations requiring rapid reversal of the anticoagulant effect of dabigatran. The indication for long-term anticoagulation was NVAF in 10 patients and deep vein thrombosis in 1 patient. Emergency situations included severe postoperative bleeding in 1 case, emergency high-bleeding-risk surgery (hip/spine surgery and neurosurgery) in 4 cases, invasive diagnostic testing (lumbar puncture) in 1 case, intracranial bleeding (pre-pontine subarachnoid and lobar intracerebral hemorrhage) in 2 cases and the decision to perform thrombolysis with rt-PA in 3 cases. Before administration of idarucizumab, laboratory coagulation tests were performed in all cases and the dTT was used to calculate the dabigatran concentration in 5 cases.

Clinical situations requiring urgent reversal of the anticoagulant effect of NOACs are expected to be relatively rare. Due to the short half-lives of NOACs, especially when renal function is normal, restoration of hemostasis can be expected within 12–24 h after the last dose intake [11]. The incidence of major bleeding events is significantly reduced with NOACs compared with vitamin K antagonists [12], and treatment discontinuation (along with supportive measures) may often be sufficient even in patients with moderate-to-severe bleeding [11, 13]. If an emergency surgical intervention or invasive procedure is required, it may in some cases be possible to postpone it until at least 12 h (and ideally 24 h) after the last NOAC dose intake to reduce the risk of bleeding complications [11].

However, there are situations such as life-threatening bleeding or urgent interventions when discontinuation of the NOAC treatment is insufficient to address the clinical need, particularly among patients with renal impairment [14]. Guidelines from the International Society on Haemostasis and Thrombosis lists the following potential indications for the use of specific reversal agents or antidotes: life-threatening bleeding, bleeding into a critical organ or closed space, prolonged bleeding despite local hemostatic measures, high risk of recurrent bleeding because of overdose or delayed clearance of the drug, and the need for an urgent intervention associated with a high risk of bleeding [15]. As some patients with NVAF present with acute ischemic stroke despite well-managed anticoagulation (1.5 per year in recent clinical trials according to Kirchhof et al. [16]) or due to treatment underdosing, thrombolysis with rt-PA may be urgently required in a NOAC-treated patient.

The clinical experience reported in this case series confirms the efficacy and ease of use of idarucizumab, and illustrates its role in improving patient safety in a variety of emergency situations requiring rapid reversal of the anticoagulant effect of dabigatran. The development and dissemination of institution-specific anticoagulation reversal protocols by multidisciplinary teams are needed to ensure timely and appropriate use of this new therapeutic option. Availability of a specific reversal agent is an important factor to be taken into consideration when choosing an anticoagulant in clinical practice.
